# Update on Optical Coherence Tomography and Optical Coherence Tomography Angiography Imaging in Proliferative Diabetic Retinopathy

**DOI:** 10.3390/diagnostics11101869

**Published:** 2021-10-11

**Authors:** Sara Vaz-Pereira, Tiago Morais-Sarmento, Michael Engelbert

**Affiliations:** 1 Department of Ophthalmology, Centro Hospitalar Universitário de Lisboa Norte, EPE—Hospital de Santa Maria, 1649-035 Lisbon, Portugal; 2 Department of Ophthalmology, Faculdade de Medicina, Universidade de Lisboa, 1649-028 Lisbon, Portugal; 3 Department of Ophthalmology, Hospital do Espírito Santo de Évora EPE, 7000-811 Évora, Portugal; tiagomsarmento@edu.ulisboa.pt; 4 Vitreous Retina Macula Consultants of New York, New York, NY 10022, USA; michael.engelbert@gmail.com; 5 LuEsther T. Mertz Retinal Research Center, Manhattan Eye, Ear and Throat Hospital, New York, NY 10065, USA; 6 Department of Ophthalmology, New York University School of Medicine, New York, NY 10016, USA

**Keywords:** diabetic retinopathy, proliferative diabetic retinopathy, retinal neovascularization, optical coherence tomography, optical coherence tomography angiography

## Abstract

Proliferative diabetic retinopathy (PDR) is a major cause of blindness in diabetic individuals. Optical coherence tomography (OCT) and OCT-angiography (OCTA) are noninvasive imaging techniques useful for the diagnosis and assessment of PDR. We aim to review several recent developments using OCT and discuss their present and potential future applications in the clinical setting. An electronic database search was performed so as to include all studies assessing OCT and/or OCTA findings in PDR patients published from 1 January 2020 to 31 May 2021. Thirty studies were included, and the most recently published data essentially focused on the higher detection rate of neovascularization obtained with widefield-OCT and/or OCTA (WF-OCT/OCTA) and on the increasing quality of retinal imaging with quality levels non-inferior to widefield-fluorescein angiography (WF-FA). There were also significant developments in the study of retinal nonperfusion areas (NPAs) using these techniques and research on the impact of PDR treatment on NPAs and on vascular density. It is becoming increasingly clear that it is critical to use adequate imaging protocols focused on optimized segmentation and maximized imaged retinal area, with ongoing technological development through artificial intelligence and deep learning. These latest findings emphasize the growing applicability and role of noninvasive imaging in managing PDR with the added benefit of avoiding the repetition of invasive conventional FA.

## 1. Introduction

Proliferative diabetic retinopathy (PDR), the more advanced stage of diabetic retinopathy (DR), remains a major cause of blindness [1,2]. The global prevalence of PDR has been projected at 7.5% [3], with around 35 million diabetic individuals suffering from PDR in 2019, whereas by 2030 and 2045, about 43 and 53 million are expected to be afflicted, respectively. Currently, eyes with PDR have a 1.5% probability at 1 year and a 3.2% probability at 2 years of succumbing to blindness, which is 3.8 and 4.0 higher than eyes with average DR at 1 year and 2 years, respectively [4]. However, due to regional differences in healthcare access, the incidence of visual impairment and blindness can reach 11.11% and 7.7%, respectively [5]. The high impact and global burden of PDR emphasize the need to continue researching diagnostic and treatment modalities [6]. Additionally, not only does the effectiveness of screening programs need to be improved [7], but also patient flow in clinics. The goal is to obtain more information with less testing and ideally without moving the patient between several machines and/or rooms, particularly with the new healthcare reality brought about by the SARS-COV-2 pandemic [6].

The hallmark of PDR is the presence of neovascularization, which is induced by global retinal ischemia [8,9,10]. Neovascular complexes (NVCs) can occur over the optic disc or within one disc diameter of the disc (NVD) or elsewhere in the retina (NVE), with severe visual loss (sVL) resulting from hemorrhagic and/or tractional complications [6,9,11].

To date, there are several recognized imaging techniques to evaluate PDR, the gold standard still being fundus fluorescein angiography (FA) [12,13,14,15]. Nevertheless, there have been significant advances in noninvasive imaging technologies, such as optical coherence tomography (OCT). OCT technology, initially described in 1991, is one of the most important recent advances in ophthalmology [16]. In 1993, for the first time, images of the human retina were obtained using OCT [17], and in medicine, ophthalmology was one of the first specialties to adopt this technique, namely to evaluate the retinal microstructure, supplementing conventional fundus examination [18]. OCT, and now OCT-angiography (OCTA), have changed the paradigm of the diagnosis of retinal disease, including DR [6,19,20]. In 2005, OCT became the standard of care for macular imaging [18], including diabetic macular edema (DME), but the first report on diabetic neovascularization was only published in 2013 [21]. Structural OCT has proven useful in PDR by allowing practitioners to visualize the NVCs, their spatial relationships and their associated vitreoretinal interface changes [6,21,22,23,24,25]. However, it has limitations, mostly in assessing disease progression and treatment response [25,26], as it cannot obtain information on the vessel structure and blood flow [27,28]. In 2014, commercial OCT angiography (OCTA) was first introduced [18]. It was developed as an extension of OCT technology, without the necessity for dye injection, as in FA [29], and allows a two- and three-dimensional noninvasive visualization of the retinal microvasculature by detecting the movement of the red blood cells within the vessels in consecutive B-scans [30,31,32,33,34,35]. Thus, OCTA is able to show the NVCs’ vascular structure and flow [6,36,37,38,39,40,41,42], and widefield OCTA (WF-OCTA) has been shown to diagnose PDR with high accuracy [6,43,44,45,46].

We previously published a systematic review on the OCT features of PDR [6], but with more publications using widefield technologies, it is appropriate to review the more important and latest findings, as in the near future, WF-OCTA will likely replace conventional FA. In the present review, we performed an update on the use of OCT, OCTA and WF-OCTA to image PDR and summarized the most recent studies.

## 2. Materials and Methods

For this narrative review, an electronic database search was performed on PubMed from 1 January 2020 until 31 May 2021 in order to find all studies assessing PDR findings using OCT and/or OCTA, specifically NVCs (NVD and NVE) and retinal nonperfusion areas (NPAs), including data concerning the foveal avascular zone (FAZ). All papers were in English, French, Spanish or Portuguese, and all publication types were included, provided they were not conference summaries and letters, complementing a previous systematic review [6]. No restrictions were placed on age, diabetes type, metabolic status or follow-up. Additional File S1 provides the detailed search strategy. All papers were screened through the title and summary by two independent reviewers, proceeding to full-text assessment if eligible. Article selection was based on themes within the scope of the review and assessment of the outcomes of interest. The articles’ reference lists were also hand-searched for additional studies. Data extraction was reviewed, with double verification reducing reporting errors.

## 3. Results

**Figure 1:**
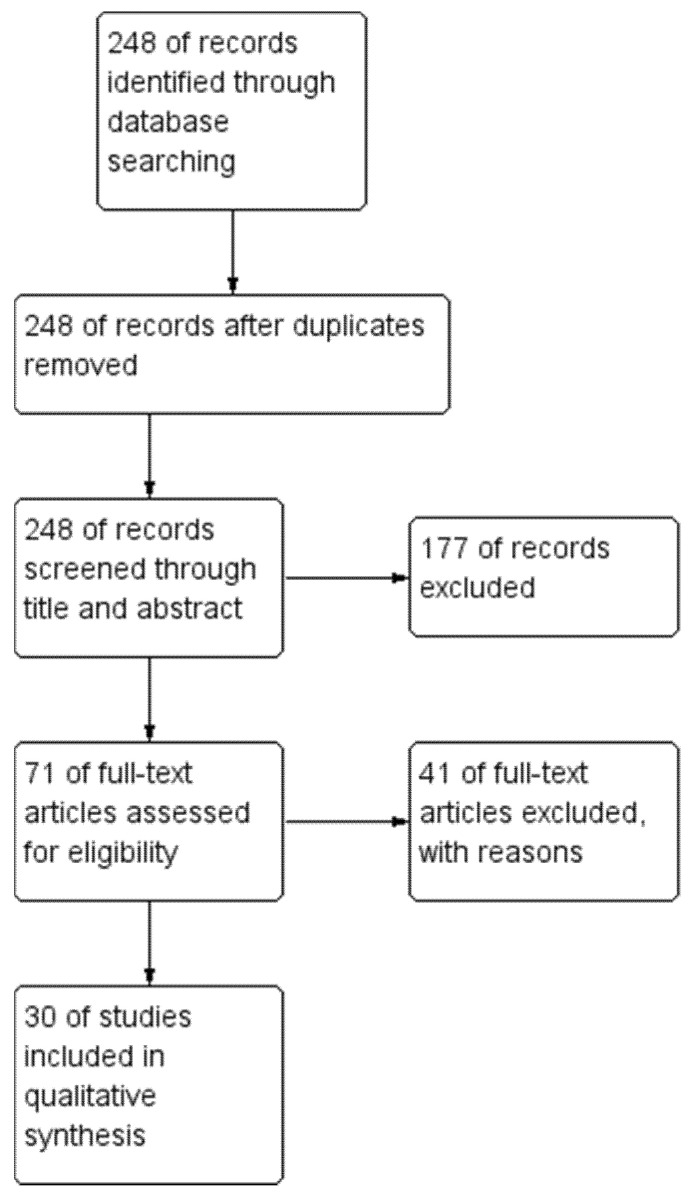
Study flow diagram.

This search recognized 248 studies, 71 were fully appraised, and 30 included in this review (Figure 1). The included studies are summarized in Additional File S2 and were mostly observational and non-comparative (nine were observational retrospective, six were observational prospective, five were reviews, four were prospective cross-sectional, two were retrospective cross-sectional, one was a prospective randomized control trial, one was a secondary analysis of previously published prospective trials, one was a retrospective analysis of prospective observational case series, and one was a case-report). Regarding the 41 excluded articles, they were excluded based on being outside the selected date interval—5, the absence of NVC or NPA results—24, not using OCT/OCTA—5, and lacking PDR data—7.

### 3.1. Update on Tomographic Features of NVC

#### 3.1.1. NVD

**Figure 2:**
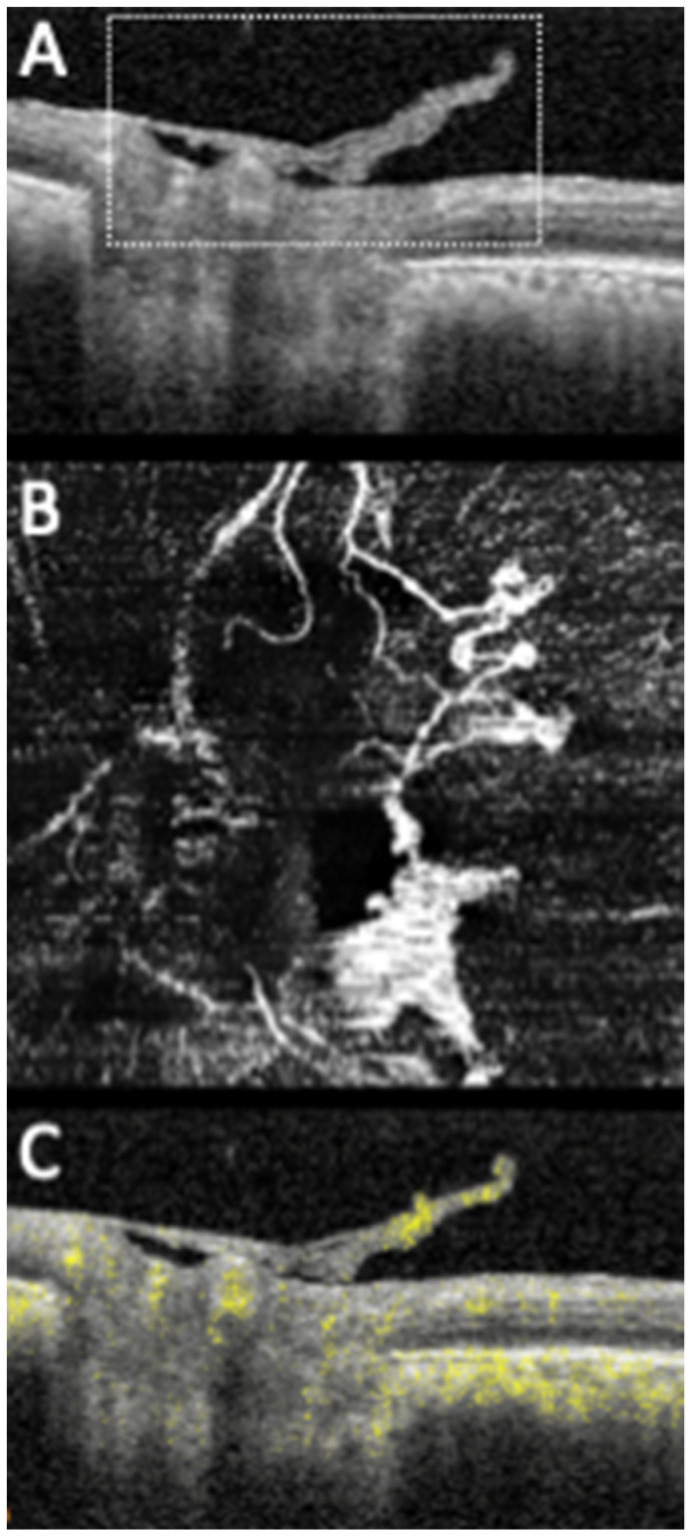
Structural SD-OCT (**A**) of a PDR patient showing a NVD protruding into the vitreous (annotated). Matching OCTA shows the filamentous irregular vessels in the en face image (**B**) with positive flow signal in the temporal buds in the B-scan (**C**), in accordance with NVD activity.

NVDs can be identified in structural OCT as tissue of medium–high reflectivity located on or protruding from the disc (Figure 2). They develop and grow along the outer aspect of the posterior hyaloid (PH) face, which serves as a scaffold. In more advanced complexes, the growth continues axially along the PH and into the peripapillary area. In some cases, there can be breaching of the PH with subsequent vitreous invasion [6,15,20,21,22,25,41,47,48]. Using OCTA, NVDs can be observed in the en face image as vascular loops or filamentous irregular new vessels located on the disc or protruding from it with positive flow signal in the corresponding B-scan (Figure 2) [6,15,20,26,27,28,36,37,39,40,41,42,43,44,45,46,47,48,49,50,51,52,53,54,55,56,57,58,59,60,61,62,63,64,65,66].

More recently, Schwartz et al. [41] reported successful employment of widefield OCT and B-scan OCTA in the detection of NVD, with clearly superior results when compared to color fundus photography (CFP). B-scan OCTA had the advantage of conveying additional information concerning the presence or absence of flow signal in the detected lesions, allowing the researchers to assess neovascular activity (Figure 2) [41]. Additionally, Khalid et al. [67] used widefield OCTA and classified NVDs into four types according to its configuration on OCTA: type 1—NVD bridging the cup, type 2—NVD with small buds, type 3—NVD flat over the inner limiting membrane (ILM) and type 4—NVD protruding into the vitreous. Furthermore, by studying the different configurations of NVD, this group found that types 1 and 2 NVD detected by WF-OCTA were undetectable by clinical examination alone, reinforcing the use of OCTA technology [67].

#### 3.1.2. NVE

**Figure 3:**
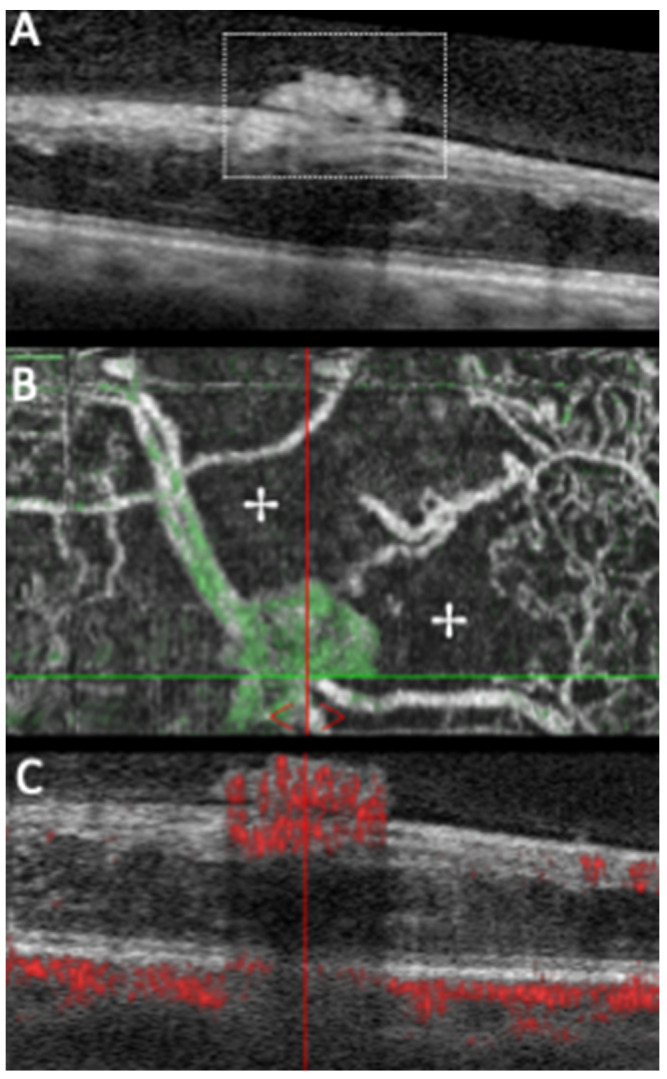
Structural SD-OCT (**A**) of a PDR case showing a flat NVE (annotated) with associated cystoid retinal edema. Same lesion OCTA shows the NVE with a globular appearance and associated areas of retinal nonperfusion (✢) in the en face image (**B**), while the B-scan (**C**) reveals positive flow signal within the inner retina, which continues into the lesion with ILM breaching, confirming an active NVE.

**Figure 4:**
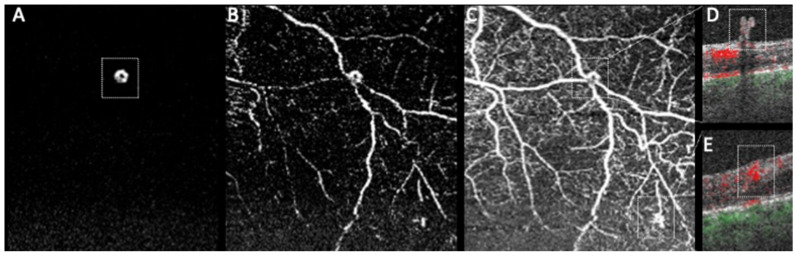
OCTA imaging of a small NVE. Note the presence of a vascular signal in the automated vitreoretinal slab (**A**), indicating the NVE (annotated). With manual segmentation (**B**), some vessels of the superficial plexus can be observed better localizing it. (**C**) is segmented between the vitreous and the inner retina and now, besides the NVE (top box), another lesion can be observed (bottom box). Corresponding B-scans show that the top lesion is indeed an NVE with ILM breaching (**D**), while the bottom lesion corresponds to an IRMA (**E**).

NVEs can be recognized in structural OCT as preretinal tissue of medium–high reflectivity that breached the ILM (Figure 3 and Figure 4). NVEs can develop at different levels of the inner retina, penetrate the ILM and can assume different morphologic patterns in relation to the retina (Figure 3 and Figure 4) [21,22,23,25,27,47,48]. Using OCTA, NVEs can be observed as irregular vessels with a positive flow signal above the ILM, which distinguishes them from other vascular lesions such as IRMA and microaneurysms (Figure 3 and Figure 4) [6,15,26,36,37,38,39,40,41,42,43,44,45,50,52,54,56,62,63,64,68,69,70,71,72,73,74,75].

Schwartz et al. [41] also successfully detected NVE and determined NVE activity in the posterior pole by combining OCT and OCTA. This strategy had an additional advantage in the presence of retinal and preretinal hemorrhages, despite their masking effect. Similarly, Belenje et al. [76] reported a particular case in which OCTA successfully detected NVCs and adjoining NPAs “hidden” under retinal hemorrhages on funduscopy.

Khalid et al. [67], in 2020, readily distinguished NVE and IRMA based on ILM penetration, with NVE presenting ILM breaching (Figure 4), as several other papers had already proposed [6,9,23,77,78]. Furthermore, this group showed that WF-OCTA detected neovascularization in 88.6% of cases compared to the 72.2% of clinical examination, proving the value of WF-OCTA as an important complementary tool to clinical examination [67,79]. The work of Cui and collaborators [80] reinforced again the significantly superior detection rates of NVE with isolated WF-OCTA when compared to ultra-widefield CFP (UWF-CFP), and found similar detection rates when comparing WF-OCTA to ultra-widefield FA (UWF-FA).

Shiraki et al. [81], using vitreoretinal interface imaging techniques in WF-OCTA, quantified the size and density of NVEs over time and concluded that the round and non-ramified growth pattern correlated with a higher retinal ischemic index, suggesting that the NVE morphology may represent a surrogate for overall ischemia in PDR. Their results also support the previous findings of non-inferiority of FA in detecting NVCs in PDR, with manual segmentation optimizing the WF-OCTA results even further.

#### 3.1.3. Additional Observations

Al-Khersan et al. [82], in 2020, looked into the challenge of any ophthalmologist identifying NVCs in WF-OCTA by comparing the performance of non-expert ophthalmologists with several training levels using WF-OCTA and FA. The agreement between graders was almost identical in the FA and WF-OCTA en face analysis, proving that ophthalmologists of all levels were able to identify NVCs in WF-OCTA [82]. However, despite the good results in NVCs with WF-OCTA, IRMAs remain a source of false positives and NVD was missed more frequently than NVE (Figure 4) [82]. Subtle smaller fronds of NVE, requiring more careful examination, were also missed more often (Figure 4) [82].

Russell et al. [83] in a longitudinal study using OCTA have recently shown that IRMAs can enlarge, elevate the inner retinal surface and breach the ILM into the vitreous cavity, strongly suggesting that IRMAs are precursors of NVC. The authors propose that the NVC process has its origin in the retinal vascular system and that IRMAs constitute a transitional stage. This transitional stage might persist for a considerable time, being possible to identify and characterize the IRMAs, while in other cases it might be short lived or very small leading to the conclusion that the new vessels arose de novo.

Pichi et al. [84] also compared WF-OCTA with UWF-FA and found no significant differences in the diagnostic accuracy of NVD and NVE, with WF-OCTA yielding sensitivity and specificity values of 100% and above 95%, respectively, for NV detection.

Kilani et al. [85] analyzed the NVC proliferation routes and their connection to the posterior vitreous status with OCTA. NVCs were found to proliferate along the posterior hyaloid membrane (72.4%), along epiretinal membranes (18.4%) and along fibrovascular membranes (9.2%). This study also compared OCTA with FA in detecting NVCs and found OCTA to be non-inferior with additional detailed information about the vessels and their topography-guided proliferation. This study further suggested that a partially detached posterior vitreous constitutes a risk factor for the development of DR and for DR progression, and that the posterior hyaloid membrane is an important factor for the development of new vessels in PDR.

Hirano et al. [86] compared WF-OCTA sensitivity to FA, particularly by assessing the vitreoretinal slab. This study revealed that WF-OCTA had a sensitivity of 73% with auto segmentation and 84% with manual segmentation for detecting NVCs (Figure 4) [86]. Regarding the discrepancy between FA and WF-OCTA, WF-OCTA false negatives (26%) were due to incorrect ILM segmentation (Figure 4), IRMAs with fluorescein leakage and diabetic papillopathy without evident neovascularization on CFP [86]. NVCs not identified on FA and identified on WF-OCTA (25%) consisted of combined small NVCs with little leakage only detectable on WF-OCTA and ILM segmentation errors generating false positives [86]. As already mentioned, Cui et al. [80] reported similar results with comparable detection rates of NVC obtained with WF-OCTA and UWF-FA.

The work by Arya et al. [87] compared relative vascular flow speed between different vascular lesions and found that while IRMA presented a turbulent, heterogenous flow with intermediate to slow speed in areas of low speed with associated ischemia, NVCs had a turbulent, heterogenous flow with intermediate to fast speed in areas with associated ischemia. Moreover, NVCs arising from arteries demonstrated high flow speed, while NVCs arising from veins had substantially lower flow speed. This group also studied flow dynamics in DR progression and found that more severe DR, such as PDR, seemed associated with overall relatively slower vascular flow in conjunction with diminished capillary density [87]. It can be hypothesized that the intermediate to slow flow speeds might be associated with retinal nonperfusion and subsequent ischemia [87]. Alam et al. [88] studied vascular complexity features in PDR, non-proliferative diabetic retinopathy and in the absence of DR. They found that PDR patients had lower vessel density values due to the increase in ischemic areas, and thus revising vascular complexity features using OCTA can be useful to differentiate PDR from NPDR.

Three studies evaluated NVCs after panretinal photocoagulation (PRP). Lupidi et al. [89] quantitively assessed NVCs on OCTA before and after PRP and showed that OCTA quantitative metrics were valid and reliable for monitoring perfusion changes in PDR treated with a laser, since both OCTA and FA changes were similar. Furthermore, it was suggested that FA sensitivity in detecting nascent or regressed NVCs might be inferior to OCTA. This group proposed a threshold of 40% reduction in both area and vascular perfusion density on OCTA as a possible biomarker for laser efficacy in PDR [89]. Vergmann et al. [90] performed similar assessments and concluded that increasing areas of retinal NVCs in OCTA were associated with PDR progression after PRP. This suggests that OCTA reflects disease activity and that it can be used to monitor PDR development and treatment response after PRP. Kim et al. [91] described, during a 12-month follow-up of PDR post-PRP, an initial decrease in OCTA perfusion metrics (perfusion density and vascular length density) during the first month post-PRP, which was followed by a continuous significant rebound increase at 12 months post-PRP. The authors postulate that the laser-induced acute retinal inflammation accounts for the initial reduction and that the long-term improved flow might be due to the peripheral NV or IRMA regression and due to the re-establishment of macular microvasculature.

**Figure 5:**
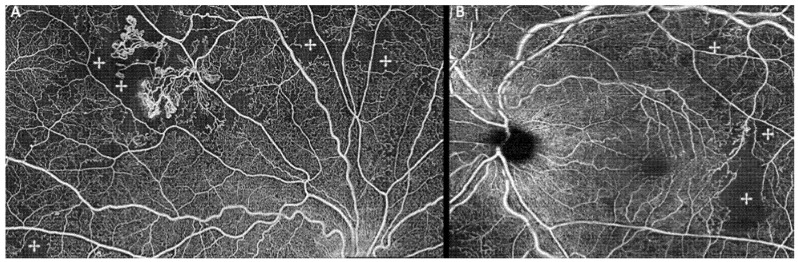
En-face SS-OCTA 8 × 8 mm montage (**A**) of a PDR patient showing areas of retinal nonperfusion (✢) mostly in the superior retina with associated retinal neovascularization. SD-OCTA 8 × 8 mm montage (**B**) of a PDR patient showing significant areas of retinal nonperfusion (✢) with a relatively well-preserved foveal avascular zone.

A study by Zhu et al. [92] searched for the optimal compromise between OCTA scanning protocol and PDR detection rates, considering that larger scanned areas might yield higher detection rates with efficiency costs on duration and difficulty of OCTA acquisition (Figure 5). This study compared the rates of detection of several features of PDR using a foveal and an optic disc centered at 6 × 6 mm and 12 × 12 mm against 15 × 9 mm montage protocols [92]. The 6 × 6 mm foveal-centered protocol had almost half the detection rate of NVCs and the 6 × 6 mm foveal-centered and optic disc-centered protocol had almost two thirds the detection range when compared to the 15 × 9 mm montage protocol, with statistically significant inferior NVC detection rates. However, despite a non-statistically significant trend favoring detection rates in the 15 × 9 mm protocol when comparing to the 12 × 12 mm foveal-centered protocol, the 12 × 12 mm foveal and optic disc-centered protocol showed similar detection rates in all PDR features to the 15 × 9 mm montage protocol [92].

Wu et al. [93], in response to the segmentation limitation challenge reported by previous studies, developed an OCTA optimization method which consists of an improved vascular connectivity analysis algorithm combined with a morphological characterization and elimination of noise and artifacts. This optimized method proved to be able to efficiently erase a significant amount of peripheric noise obtaining clean vascular networks in NVCs within the OCTA scans. It was also able to diminish near-point noise and totally negate artificial noise, leading to much more precise vascular length and width measurements.

### 3.2. NPAs

NPAs represent ischemic areas and can be observed as areas of absent flow signal or sparse capillary density (Figure 5) [6,19,42,49,51,53], and often their visualization in OCTA was superior to FA [36,51,53,79]. In some studies, NPAs could be quantified [43,44,69]. PDR patients were found to have a significant lower capillary density and increased and irregular FAZ compared to non-PDR patients (Figure 5) [57,69]. Additionally, IRMA and NVCs have been often associated with NPAs (Figure 5) [6,38,39,42,44,49,50,51,52,53,54,74,94,95,96], reinforcing the concept that global retina ischemia upregulates VEGF and leads to abnormal angiogenesis [6,38,42,79].

Uchitomi et al. [97] studied the retinal layer of NPAs using WF-OCTA and classified NPAs in deep NPA (dNPA) if noted on the deep capillary plexus (DCP) layer, and superficial NPA (sNPA) if observed on the superficial capillary plexus (SCP) layer. WF-OCTA identified more dNPAs than matching sNPAs overall and in each quadrant [97]. Considering foveal rings, the outer ring (6–10 mm) showed higher dNPAs than the intermediate (3–6 mm) and inner rings (1–3 mm) in inferior, superior and temporal quadrants, while no difference was shown in the nasal quadrant [97]. Regarding sNPAs in foveal rings, sNPAs were more frequent in outer than in intermediate rings in inferior and temporal subfields [97]. These results also suggest a higher incidence of NPAs in temporal compared to nasal quadrants [97].

Um et al. [98] assessed the advantages of OCTA in studying FAZ and consequently diabetic macular ischemia (DMI). The results show a greater FAZ area and lesser vessel density (VD) with increasing DR severity [79,98]. The DCP FAZ area remained superior to the SCP FAZ area [98]. VD declined on DCP, although remaining higher than on SCP. However, in severe NPDR and PDR DCP VD is lower than SCP VD, suggesting a higher deterioration of DCP VD with DR progression [98].

Wang et al. [99] suggested using UWF-OCTA to classify DR based on the percentage of NPA within the field of view. As expected from previous publications [38,39,44,49,50,51,52,53,54,57,69,74,94,95,96], the mean ratio of nonperfusion was highest in PDR, intermediate in NPDR and smallest in the absence of DR [99]. The field of view comparison with ROC analysis showed the highest optimal sensitivity and specificity values with 50°–100° field of view, meaning an ultra-wide field [99]. Furthermore, the results provide a cut-off value of 21.2% of nonperfusion ratio to identify NPDR and a value of 31.6% to distinguish NPDR from PDR [99].

Ashraf et al. [100] analyzed OCTA metrics in PDR with and without predominantly peripheral lesions (PPL). This study found that reduction in flow density did not seem to change with increasing DR severity in PDR with PPL, while it decreased with increasing DR severity in PDR without PPL [100].

Regarding treatment options, Alagorie et al. [101] studied the role of OCTA in monitoring ischemia in response to anti-VEGF therapy for 12 months in PDR without DME, whether on monthly or quarterly injection. OCTA showed no change in mean macular VD and flow area during the 12 months and no difference between the monthly and quarterly injection cohorts [101]. On the other hand, Russell et al. [102] focused on NPA changes in OCTA after PRP for 12 months and found that there were no significant changes in NPA immediately following and for up to 1-year after treatment. Considering the OCTA findings of stable NPAs and VD under treatment, it was postulated that the underlying mechanism for the observed progressive retinal thinning in PDR cannot be progressive nonperfusion and ischemia [101]. Furthermore, the results of Ashraf et al. [100] seem to suggest that in each eye with DR, there is a spectrum of nonperfusion ranging from posterior to peripheral nonperfusion predominance, with several grades in between.

## 4. Discussion

OCT, and now OCTA, have changed the paradigm of the diagnosis of retinal disease, including DR [6,19,20]. OCT, while traditionally used to evaluate DME, has been shown to provide important information in PDR. In 2020, OCT technology had already achieved a level of proficiency capable of accurately detecting and characterizing NVCs, NPAs and disease activity with recognized added value of WF and OCTA in disease staging and monitoring [6]. Namely, with OCT, it is possible to visualize the retinal layers in detail and to distinguish between NVC and IRMAs, and there is less obscuration than FA when there is associated fibrosis and hemorrhaging [6,41,42,79,83,103,104]. In both NVE and NVD, the OCTA flow signals found within the hyperreflective material indicate disease activity and correlate with the OCTA en face image [6,67,72,79]. OCTA is particularly useful and superior to OCT in monitoring treatment response, being able to pinpoint regression, reactivation and resistance to treatment [6,41,79].

The ability to image the entire posterior pole simultaneously is a great advantage of WF-OCTA [6,41]. Additionally, WF-OCTA images can be readily obtained in bilateral PDR, whereas in FA it is difficult to attain the earliest phase bilaterally [82]. WF-OCTA can better differentiate between IRMA, diabetic papillopathy and neovascularization than FA, due to the three-dimensional information [83,84,86,97,104,105]. UWF-OCTA can also play a role in the anti-VEGF treatment of PDR by providing more supportive information for management decisions at each visit, similar to when treating DME [6,91,99,103]. Specifically, WF-OCTA may be helpful in targeted or conventional panretinal photocoagulation, as previous studies already demonstrated the non-inferiority of extended targeted PRP to conventional PRP in PDR [89,90,91,99,106]. Based on all these results comparing FA and WF-OCTA in detecting PDR, we agree with the adoption of WF-OCTA as a stand-alone imaging modality for diagnosing PDR in clinical practice without the risk of increasing false positives and overtreatment or false negatives and undertreatment [6,41,80,82,84,85,86,91,97,99,105,106].

Nevertheless, it should be noted that for a proper assessment, correct segmentation is necessary, and often there is improvement in NVC recognition with manual segmentation [79,81,86,93,103,104]. In the future, the accuracy of NVC detection in vitreoretinal slabs might increase with improvement of auto segmentation, deep learning and artificial intelligence [81,86,107,108,109,110,111]. IRMAs seem to generate higher false positive rates due to the retinal slab image, despite the vitreoretinal slab image not showing extension into the vitreous cavity [82]. Additionally, after some debate, recent studies demonstrate that IRMAs are definite precursors of NVE [83].

NVD can be missed more often than NVE, probably because in some instances, it may appear to be in the retinal plane bridging the potential space of the optic cup [6,82].

Another important issue that remains from all these different studies [41,67,80,82,86,92,97,99,106] with different imaging protocols is the optimal acquisition protocol so as to have a balance between the scanned area and lesion detection. It seems the protocol with best detection rate was the 12 × 12 mm angiography images centered on the fovea and optic disc, being an interesting non-inferior alternative to 15 × 9 mm montage images [92,103,104]. However, some software devices also allow montages, which also increases the scanned area [6,42,103].

Regarding the remaining limitations, the large number of different OCTA devices commercially available, the motion artifacts, the media opacities, the need for particular software for NPA, FAZ and VD analysis and the acquisition of larger scans to hasten image acquisition with consequent reduction in vascular resolution constitute active challenges to be addressed in the future research into PDR detection and management.

We would also like to acknowledge that in this narrative review, we only performed the search in one database, but used a comprehensive search strategy as used in systematic reviews and also hand-searched the articles’ reference lists for additional studies, so we believe our search is accurate and reports the most recent studies on this topic.

The present study, by assessing the most recent research using OCT and OCTA in PDR, shows robust evidence of WF-OCTA being non-inferior to UWF-FA and UWF-CFP in detecting NVCs and NPAs and of the validation of WF-OCT and WF-OCTA in PDR diagnosis and management. It also suggests that the currently available WF-OCTA protocols may be adequate enough considering the spatial distribution of PDR lesions (up to 50°–100°). Nonetheless, none of these findings stand without correct segmentation and optimized scanned area, which will be subject to further research and improvement in the upcoming years.

## 5. Conclusions

Detection and characterization of diabetic neovascularization using noninvasive imaging can be useful in understanding the pathogenesis and aid in the diagnosis, monitoring and treatment of PDR, including in surgical planning and even during surgery, if intraoperative OCT is available. Additionally, it can obviate the need for invasive conventional FA, which cannot be reasonably performed at every patient visit. More recently, OCTA and WF-OCTA have had an increasing role in describing retinal nonperfusion areas, microvascular abnormalities and particularly in detecting neovascular complexes aiding in the management of DR.

## Data Availability

The data are available from the corresponding author upon reasonable request.

## Supplementary Materials

The following are available online at https://www.mdpi.com/article/10.3390/diagnostics11101869/s1, Additional File S1—Search strategy. Additional File S2—Summary of the included studies.
