# Supplementary material for: Update on Optical Coherence Tomography and Optical Coherence Tomography Angiography Imaging in Proliferative Diabetic Retinopathy
Source: Diagnostics (Basel). 2021 Oct 11;11(10):1869. doi: 10.3390/diagnostics11101869 (PMC8535055; doi:10.3390/diagnostics11101869)
Supplement: Supplementary file 1 [file diagnostics-11-01869-s001.zip › Additional File 1 - Search Strategy.pdf]

Search Strategy	
**Method No.**	**Search terms**
PubMed	
1.	Tomography, Optical Coherence [Majr]
2.	Proliferative Diabetic retinopathy [Majr]
3.	2020/1/1:2021/5/31 [pdat]
4.	English [lang]
5.	French [lang]
6.	Portuguese [lang]
7.	Spanish [lang]
8.	4 or 5 or 6 or 7
9.	1 and 2 and 3 and 8
PubMed	
1.	Optical coherence tomography [All Fields]
2.	OCT [All Fields]
3.	1 or 2
4.	Diabetic retinopathy [All Fields]
5.	DR [All Fields]
6.	Diabet* [All Fields]
7.	Retinopathy [All Fields]
8.	6 and 7
9.	4 or 5 or 8
10.	Neovascularization [All Fields]
11.	2020/1/1:2021/5/31 [pdat]
12.	English [lang]
13.	French [lang]
14	Portuguese [lang]
15.	Spanish [lang]
16.	12 or 13 or 14 or 15
17.	3 and 9 and 10 and 11 and 16
