# Supplementary material for: Update on Optical Coherence Tomography and Optical Coherence Tomography Angiography Imaging in Proliferative Diabetic Retinopathy
Source: Diagnostics (Basel). 2021 Oct 11;11(10):1869. doi: 10.3390/diagnostics11101869 (PMC8535055; doi:10.3390/diagnostics11101869)
Supplement: Supplementary file 1 [file diagnostics-11-01869-s001.zip › Additional File 2 - Summary of included studies.pdf]

Table S1. Summary of included studies

Study ID[ref]	Study Center	Study Design	Included sample	Imaging technique	Outcomes
**Alagorie 2020[101]**	USA	Post hoc analysis of a randomized clinical trial	32 eyes with PDR	OCTA	OCTA in monitoring ischemia in response to anti-VEGF therapy for 12 months in PDR without DME whether on monthly or quarterly injection.<br>OCTA showed no change in mean macular VD and flow area and no difference between the injection cohorts.
**Alam 2021[88]**	USA	Prospective cross-sectional	20 control patients<br>60 NPDR<br>56 PDR	SD-OCT<br>OCTA	Study of vascular complexity features in PDR, NPDR and in the absence of DR. Vessel complexity index and blood vessel tortuosity were the most sensitive in differentiating NPDR and PDR patients.
**Al-khersan 2021[82]**	USA	Retrospective case-series	47 eyes from 24 patients with NPDR and PDR	WF-SS-OCTA<br>WF-FA	Compared the performance of non-expert ophthalmologists with different training levels using WF-OCTA and FA. Agreement between graders was almost identical on the FA and WF-OCTA en face analysis, proving that ophthalmologists of all levels were able to identify NVCs in WF-OCTA.<br>IRMAs were a source of false positives and NVD were missed more frequently than NVE.
**Amato 2020[104]**	Italy<br>USA<br>Greece	Review	DR	WF-OCTA	WF-OCTA offers advantages over traditional imaging in the study of NPA and NVCs.<br>WF-OCTA artifacts in DR patients fall into 3 categories: 1) systemic artifacts, 2) image processing errors and 3) motion artifacts.
**Arya 2021[87]**	USA	Retrospective cross-sectional	13 NPDR<br>9 PDR eyes	SS-OCTA	Descriptive flow analysis of the retinal vascular features of DR. IRMA had turbulent, heterogenous flow with intermediate to slow speed in areas of low speed with associated ischemia. NVCs had turbulent, heterogeneous flow with intermediate to fast speed in areas with associated ischemia. PDR was associated with overall relatively slower vascular flow in conjunction with diminished capillary density.
**Ashraf 2020[100]**	USA<br>Egypt	Prospective cross-sectional	254 NPDR<br>98 severe NPDR/PDR eyes	OCTA<br>UWF-CFP	Reduction of flow density did not seem to change with increasing DR severity in PDR with PPL, while it decreased with increasing DR severity in PDR without PPL.
**Belenje 2020[76]**	India	Case-report	1 eye with PDR	WF-SS-OCTA	WF-OCTA successfully detected NVCs and adjoining NPA “hidden” under retinal hemorrhages on funduscopy.
**Chua 2020[79]**	Singapore<br>Austria<br>Switzerland	Review	DR	FA<br>OCT<br>OCTA	OCTA analysis of vascular changes qualitatively in varying stages of DR has improved the understanding of the pathophysiology of DR.<br>Potential to be an objective tool in monitoring DR progression.
**Cui 2021[80]**	USA	Prospective observational	20 control patients<br>57 NPDR<br>75 PDR	UWF-CFP<br>UWF-FA<br>WF-SS-OCTA	WF-SS-OCTA was superior to UWF-CFP in detecting IRMAs and NVCs.<br>WF SS-OCTA and UWF-FA had comparable diagnostic efficacy in differentiating NDR, PDR and NPDR.<br>OCTA plus UWF-CFP had a detection rate of all DR lesions identical to UWF-FA.
**He 2020[72]**	China	Prospective pilot study	44 eyes with PDR	FA<br>OCTA	Longitudinal changes of NVE using OCTA after PRP or combination of intravitreal conbercept plus PRP. This combination was more effective than PRP monotherapy in NVE regression.

Table S1. Summary of included studies

**Hirano 2020[86]**	Japan	Retrospective study	42 eyes with PDR	WF-SS-OCTA<br>FA<br>CFP	Comparison between WF-OCTA and FA. WFOCT had a sensitivity of 73% with auto segmentation and 84% with manual segmentation for detecting NVCs. The discrepancies were due to incorrect ILM segmentation, IRMAs with fluorescein leakage and diabetic papillopathy without evident neovascularization on CFP.
**Khalid 2021[67]**	UK<br>Egypt<br>Austria	Retrospective observational case series	22 eyes with severe NPDR<br>57 eyes with PDR	WF-OCTA	WF-OCTA classification of NVDs into 4 types according to its configuration: type 1 – NVD bridging the cup, type 2 – NVD with small buds, type 3 – NVD flat over ILM and type 4 – NVD protruding into the vitreous.<br>Types 1 and 2 NVD detected by WF-OCTA were undetectable by clinical examination alone, reinforcing the use of OCTA technology.
**Kilani 2021[85]**	Germany	Prospective observational study	42 eyes with PDR	OCTA<br>FA	Characterization of NVCs.<br>OCTA was non-inferior to FA in detecting NVCs.<br>The posterior hyaloid membrane is an important factor for development of NVCs in PDR.
**Kim 2021[91]**	South Korea	Retrospective observational study	27 eyes with PDR	SD-OCT<br>SS-OCTA<br>FA	Evaluation of quantitative changes in microvascular parameters after PRP.<br>Decrease in OCTA perfusion metrics during the 1st month post-PRP which was followed by a continuous significant rebound increase at 12 months post-PRP.
**Lupidi 2020[89]**	Italy<br>France<br>USA	Prospective observational	15 eyes with PDR	OCTA<br>FA	OCTA quantitative metrics were valid and reliable for monitoring perfusion changes in PDR treated with laser, with OCTA and FA changes being similar.<br>FA sensitivity in detecting nascent or regressed NVCs might be inferior to OCTA.<br>40% reduction in both area and vascular perfusion density on OCTA might be a possible biomarker for laser efficacy in PDR.
**Markan 2020[105]**	India	Review	DR	FA<br>OCT<br>OCTA	WF-OCTA and flow overlay enable to distinguish between NVE and IRMA.<br>OCTA is useful for MAs, IRMAs, NPA and NVCs even before clinically seen clinically on fundus photography.<br>WF-OCTA has been compared with UWF-FA in patients with DR with recent observational studies showing similarity in detecting NVCs.<br>WF-OCTA allows the detection of subtle changes in NVCs after treatment, predicting the future course of these lesions.
**Pichi 2020[84]**	UAE<br>USA	Prospective cross-sectional	82 eyes with PDR	UWF-FA<br>UWF-CP<br>WF-OCTA	WF-OCTA and UWF-FA revealed different diagnostic accuracy values for NVD and NVE, with WF-OCTA yielding sensitivity and specificity values of 100% and above 95% for NVC detection, respectively.
**Russell 2020[83]**	USA	Retrospective analysis of prospective, observational case series	20 patients with PDR	WF-SS-OCTA<br>UWF-FA	Longitudinal study of IRMAs.<br>IRMAs can enlarge, elevate the inner retinal surface and breach the ILM into the vitreous cavity, strongly suggesting that IRMAs are precursors to NVCs.
**Russell 2020[102]**	USA	Prospective, observational, consecutive case series	20 eyes with PDR	WF-SS-OCTA<br>UWF-CFP<br>UWF-FA	Assessment of NPA before and after PRP.<br>There were no significant changes in NPA in WF-OCTA immediately following and for up to 1-year after PRP.

Table S1. Summary of included studies

**Schwartz 2020[41]**	UK	Retrospective, observational case series	47 eyes with PDR	OCTA	OCT/OCTA to monitor PDR.<br>Structural OCT had the best detection rate for NVCs and B-scan OCTA had the most potential disease monitoring after treatment.
**Shiraki 2021[81]**	Japan	Retrospective observational	25 eyes with PDR	WF-SS-OCTA<br>UW-FA	Vitreoretinal interface imaging techniques in WF-OCTA quantified the development of NVEs in PDR and evaluated the factors involved in NVE growth speed.<br>WF-OCTA was non-inferior to FA in detecting NVCs in PDR, with manual segmentation optimizing further the WF-OCTA results.
**Uchitomi 2020[97]**	Japan	Retrospective, observational case series.	104 eyes with PDR	WF-SS-OCTA	Characterized the NPA in posterior pole in PDR.<br>WF-OCTA identified deeper NPA than matching superficial NPA overall and in each quadrant.
**Um 2020[98]**	South Korea	Retrospective, observational case series.	85 eyes with PDR	OCTA	Greater FAZ area and lesser VD with increasing DR severity.<br>In severe NPDR/PDR, DCP VD is lower than SCP VD, suggesting a higher deterioration of DCP VD with DR progression.
**Vaz-Pereira 2020[42]**	Portugal<br>USA	Retrospective case series	23 eyes with PDR	FA<br>SD-OCT<br>OCTA	OCTA is useful to image NVCs, IRMAs and NPA in eyes with DR.<br>The detection of flow in NVCs using OCTA has potential to obviate the need for additional more invasive dye-based imaging.
**Vaz-Pereira 2020 [6]**	Portugal	Review	PDR	FA<br>OCT<br>OCTA<br>WF-OCTA	OCT was useful in detecting NVCs, in characterizing disease activity and response to laser and/or anti-VEGF therapies.<br>The absence of posterior vitreous detachment seemed determinant for neovascular growth with the posterior hyaloid acting as a scaffold.<br>OCTA allowed a more detailed characterization of NVCs, associated NPA and disease activity, allowing the quantification of neovessel area and flow index.<br>Changes in OCTA blood flow signal following local therapies did not necessarily correlate with structural regression.<br>WF-OCTA was highly sensitive in the detection of PDR, adding value to disease staging and monitoring.
**Vergmann 2020[90]**	Denmark	Prospective randomized controlled trial	53 eyes with PDR	SD-OCT<br>OCTA	Increasing areas of retinal NVCs in OCTA were associated with PDR progression after PRP. OCTA seems to reflect disease activity and it can be used to monitor PDR development and treatment response after PRP.
**Wang 2020[99]**	USA	Retrospective cross-sectional	17 eyes without DR<br>19 eyes with NPDR<br>24 eyes with PDR	WF-SS-OCTA	WF-OCTA can classify DR based on the percentage of NPA within the field of view.<br>Mean ratio of nonperfusion was highest in PDR.<br>The field of view comparison with ROC analysis showed highest optimal sensitivity and specificity values with 50-100° field of view.
**Wang 2021[103]**	USA	Review	PDR	WF-CFP<br>WF-SS-OCTA<br>UWF-FA	WF-OCTA is at least non-inferior to UWF-FA and WF-CFP at detecting NVE and peripheral non-perfusion, if not more sensitive.<br>WF-SS-OCTA is increasingly used to diagnose and monitor DR.

Table S1. Summary of included studies

**Wu 2021[93]**	China	Prospective, comparative, observational clinical trial	14 eyes with PDR	OCTA	OCTA optimization method consisting of an improved vascular connectivity analysis algorithm combined with morphological characterization and elimination of noise and artifacts.
**Zhu 2020[92]**	USA	Prospective observational study	23 eyes without DR<br>73 eyes with NPDR<br>80 eyes with PDR	UWF-CFP<br>UWF-FA<br>WF-SS-OCTA	Compared rates of detection of several features of PDR using foveal and optic disc centered 6x6 mm and 12x12 mm against 15x9 mm montage protocols.<br>The 12x12 mm protocols showed similar detection rates in all PDR features to the 15x9 mm protocol and were better than the 6x6 mm protocol.

CFP color fundus photography, *DCP* deep capillary plexus, *DME* diabetic macular edema, *DR* diabetic retinopathy, *FA* fluorescein angiography, *FAZ* foveal avascular zone, *ILM* inner limiting membrane, *IRMA* intraretinal microvascular abnormalities, *MA* microaneurysm, *NPA* retinal nonperfusion areas, *NDPR* non-proliferative diabetic retinopathy, *NVC* neovascular complex, *NVD* neovascularization of the disc, *NVE* neovascularization elsewhere, *OCT* optical coherence tomography, *OCTA* optical coherence tomography angiography, *PDR* proliferative diabetic retinopathy, *PPL* predominantly peripheral lesions, *PRP* panretinal photocoagulation, *SCP* superficial capillary plexus, *SD-OCT* spectral-domain optical coherence tomography, *SS-OCT* swept-source optical coherence tomography, *SS-OCTA* swept source optical coherence tomography angiography, *VD* vessel density, *VEGF* vascular endothelial growth factor, *UWF* ultra-widefield, *WF* widefield
